# Supplementing Inosine to Blood Collection Tubes Adds a Glycolytic Inhibitory Effect

**DOI:** 10.1111/1753-0407.70144

**Published:** 2025-08-20

**Authors:** Yukio Kume, Motohiro Ohkubo, Naru NaKatuka, Sayaka Aritake‐Okada, Teruhiko Yoshida, Hideaki Isago, Makoto Kurano

**Affiliations:** ^1^ Department of Clinical Laboratory The University of Tokyo Hospital Tokyo Japan; ^2^ Graduate School of Health and Social Services, Saitama Prefectural University Saitama Japan; ^3^ Department of Clinical Laboratory Medicine Graduate School of Medicine, University of Tokyo Tokyo Japan

To the Editor,

At present, sodium fluoride (NaF)‐supplemented tubes are used as usual blood collection tubes that can measure glucose and glycated hemoglobin (HbA1c) simultaneously. However, blood collection with NaF tubes show a decrease in glucose levels at room temperature within 4 h after blood collection [1]. The American Diabetes Association (ADA) guidelines recommend immersion of blood samples collected with heparin‐lithium (Hp‐Li) in ice water within 30 min or the use of tubes containing NaF and citrate buffer (FC) [2]. However, immediate immersion in ice water is difficult in clinical practice and citric acid is difficult to dissolve and is not suitable for HbA1c measurements because of hemolysis. Therefore, we aimed to develop blood collection tubes that are easier for clinical use than FC tubes and minimize blood glucose decline, compared with NaF tubes.

We collected 2 mL of whole blood each tube. Immediately after blood collection, each tube was agitated on a mix rotator at 3000 rpm (1470 g) for 5 min and then stored under storage conditions until immediately before measurement. The aliquoted blood collection tubes were stored at room temperature (25°C) or refrigerated (4°C). Then, the aliquoted whole blood was plasma‐separated by centrifugation immediately and 2, 4, 24, and 48 h after collection. Glucose was measured immediately after separation, as well as HbA1c.

Changes in blood glucose levels in the NaF, FI, and FC tubes are shown in Figure 1A–D. Blood glucose changes were significantly attenuated to a greater degree in FI tubes than in NaF tubes at 4°C and 25°C conditions 4 and 24 h after sampling, whereas the blood glucose‐preserving abilities of FI tubes were significantly inferior to those of FC tubes, except the case when stored at 4°C for 4 h. Regarding the criteria required from ADA (within 6.1% of the baseline blood glucose levels), the storage of FI blood collection tubes at 4°C did not reduce blood glucose levels by > 6.1%, which is the criteria required from ADA, in any sample up to 48 h, whereas the storage of FI blood collection tubes at room temperature reduced blood glucose levels by > 6.1% in 1 of 10 cases after 24 h (Figure S2), which were much superior to the NF tubes (Figures S1 and S2).

**FIGURE 1:**
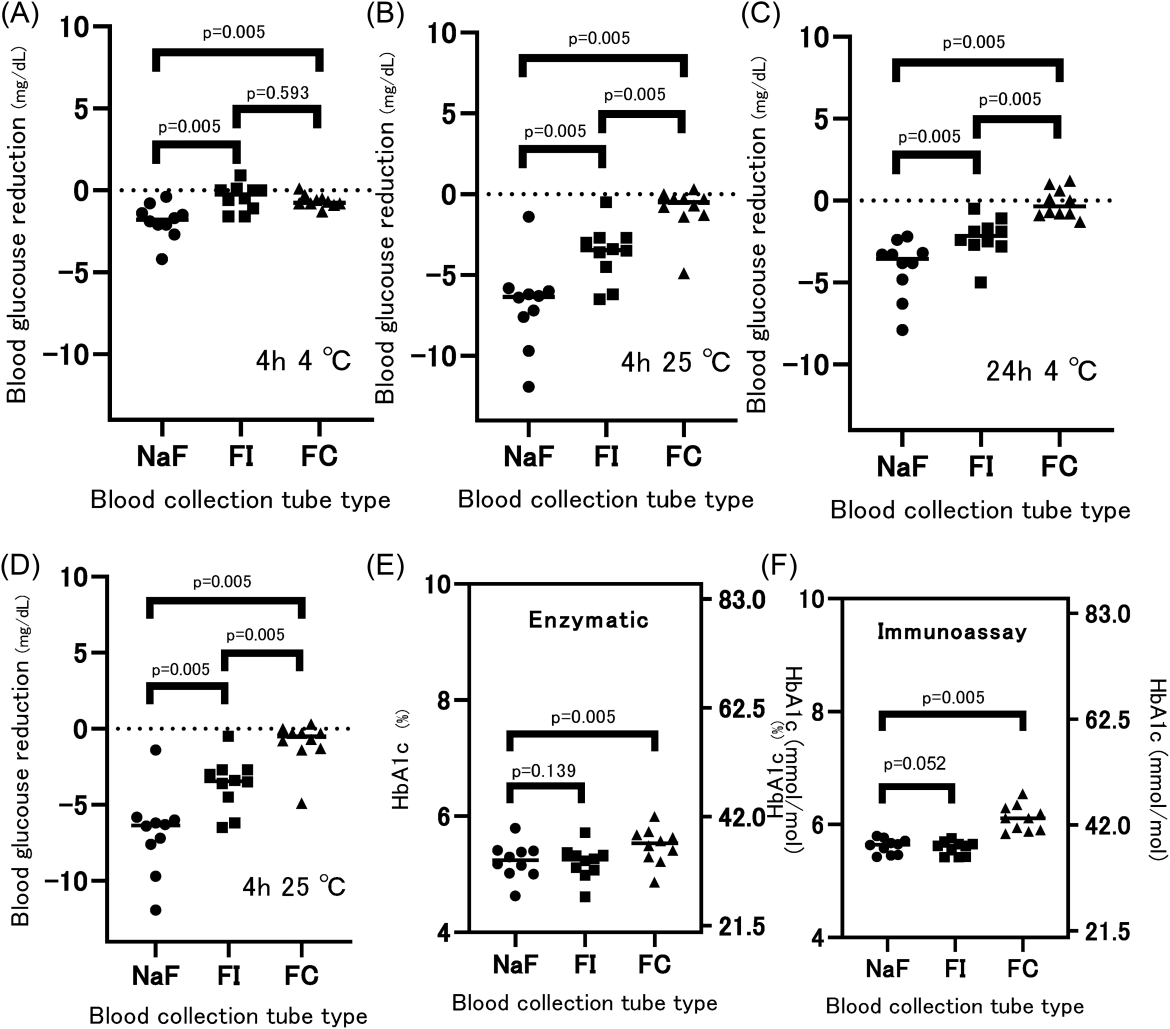
Difference in changes in blood glucose concentration and HbA1c among various blood collection tubes. Blood samples were taken from 10 volunteers, and blood glucose decline was measured in various blood collection tubes. Closed circles represent NaF tubes containing NaF, close boxes represent FI tubes containing NaF and inosine, and closed triangles represent FC tubes containing NaF and citric acid. (A, B) Decrease in blood glucose concentrations were evaluated at 4°C after 4 h (A) or 24 h (B) (*n* = 10). (C, D) Decrease in blood glucose concentrations were evaluated at 25°C after 4 h (C) or 24 h (D) (*n* = 10). (E, F) Enzymatic and immunoassay HbA1c levels were measured in various blood collection tubes. Conversion formula from NGSP to IFCC: IFCC value (mmol/mol) = 10.93 × NGSP value‐23.52. FC, FC tubes containing NaF and citric acid; NaF, NaF tubes containing NaF; FI, FI tubes containing NaF and inosine. The *p* values were calculated using the Wilcoxon signed rank test.

In addition, the enzymatic and immunoassay methods showed a significant difference with a *p* value of 0.005 in FC blood tubes, whereas no significant difference was observed for the FI blood tubes (Figure 1E,E). Concordantly, no significant difference in hemolytic Hb levels was observed in the NaF and FI tubes, but not in FC tubes, compared with Hp‐Li tubes (Figure S4).

Regarding the mechanism, although the detail mechanisms will be published somewhere else, the inhibitory effects on ATP and glucose uptake decline in erythrocytes were considered. We believe that inosine addition to the NaF blood collection tube would contribute to measuring the blood glucose levels exactly, without disturbing HbA1c measurements.

## Author Contributions

Y.K. and M.K. designed the study. M.K. supervised the study. Y.K., M.O., N.N., and M.K. carried out the research. Y.K. wrote the manuscript. S.A.‐O., T.Y., H.I., and M.K. reviewed the manuscript draft. All authors accept responsibility for the entire content of this manuscript and have approved its submission.

## Ethics Statement

This study was approved by the ethics committee of the Graduate School of Medicine at the University of Tokyo (Approval no. 2020063NI).

## Consent

Healthy participants provided written informed consent.

## Conflicts of Interest

The authors declare no conflicts of interest.

## Supporting information


**Figure S1:** jdb70144‐sup‐0001‐FigureS1.tif.

**Figure S2:** jdb70144‐sup‐0002‐FigureS2.tif.

**Figure S3:** jdb70144‐sup‐0003‐FigureS3.tif.

**Figure S4:** jdb70144‐sup‐0004‐FigureS4.tif.
