# Supplementary figures and images for: Supplementing Inosine to Blood Collection Tubes Adds a Glycolytic Inhibitory Effect
Source: J Diabetes. 2025 Aug 20;17(8):e70144. doi: 10.1111/1753-0407.70144 (PMC12365536; doi:10.1111/1753-0407.70144)

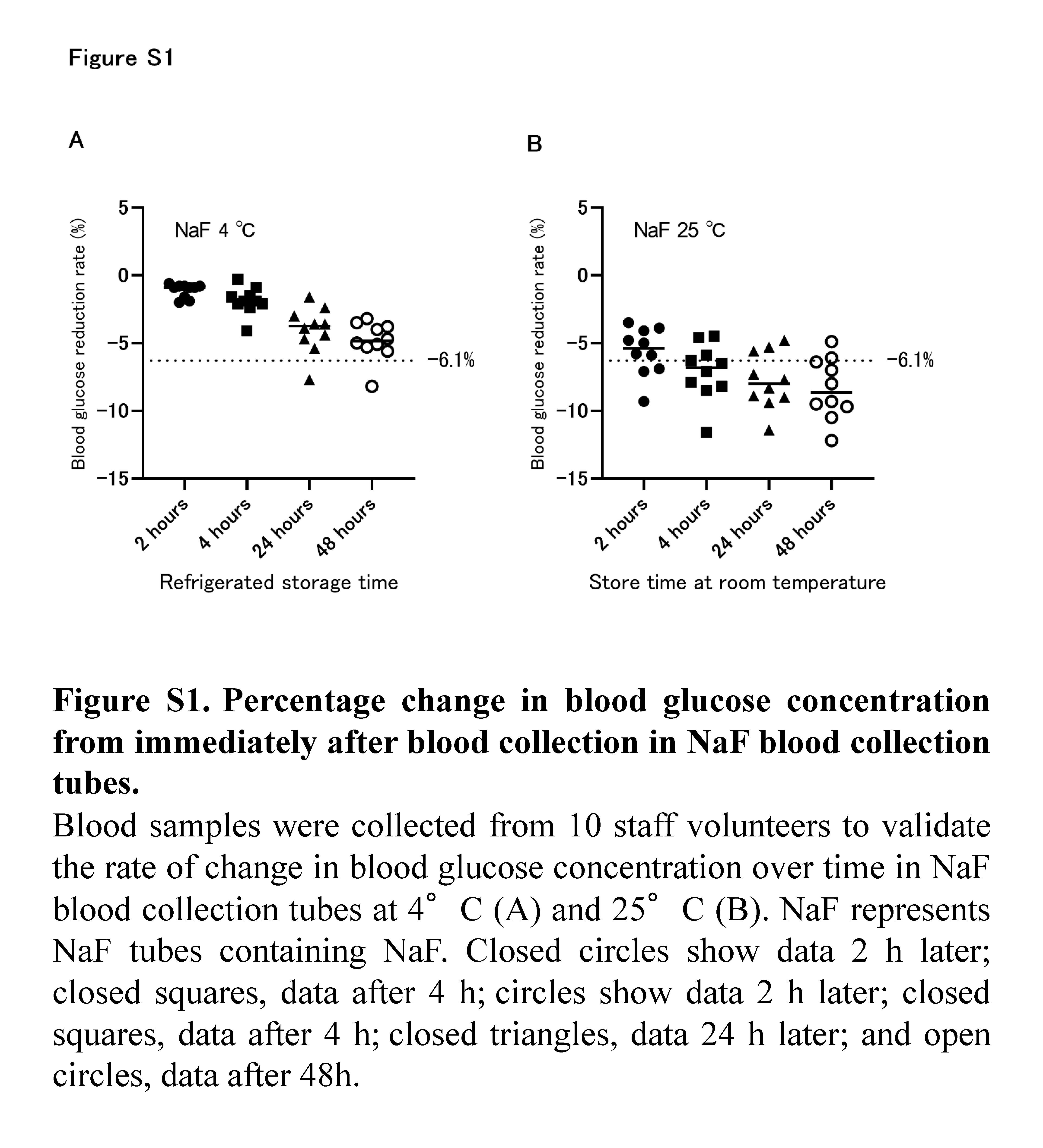

Supplement: Supplementary file 1 — Figure S1: jdb70144‐sup‐0001‐FigureS1.tif. [file JDB-17-e70144-s002.tif]

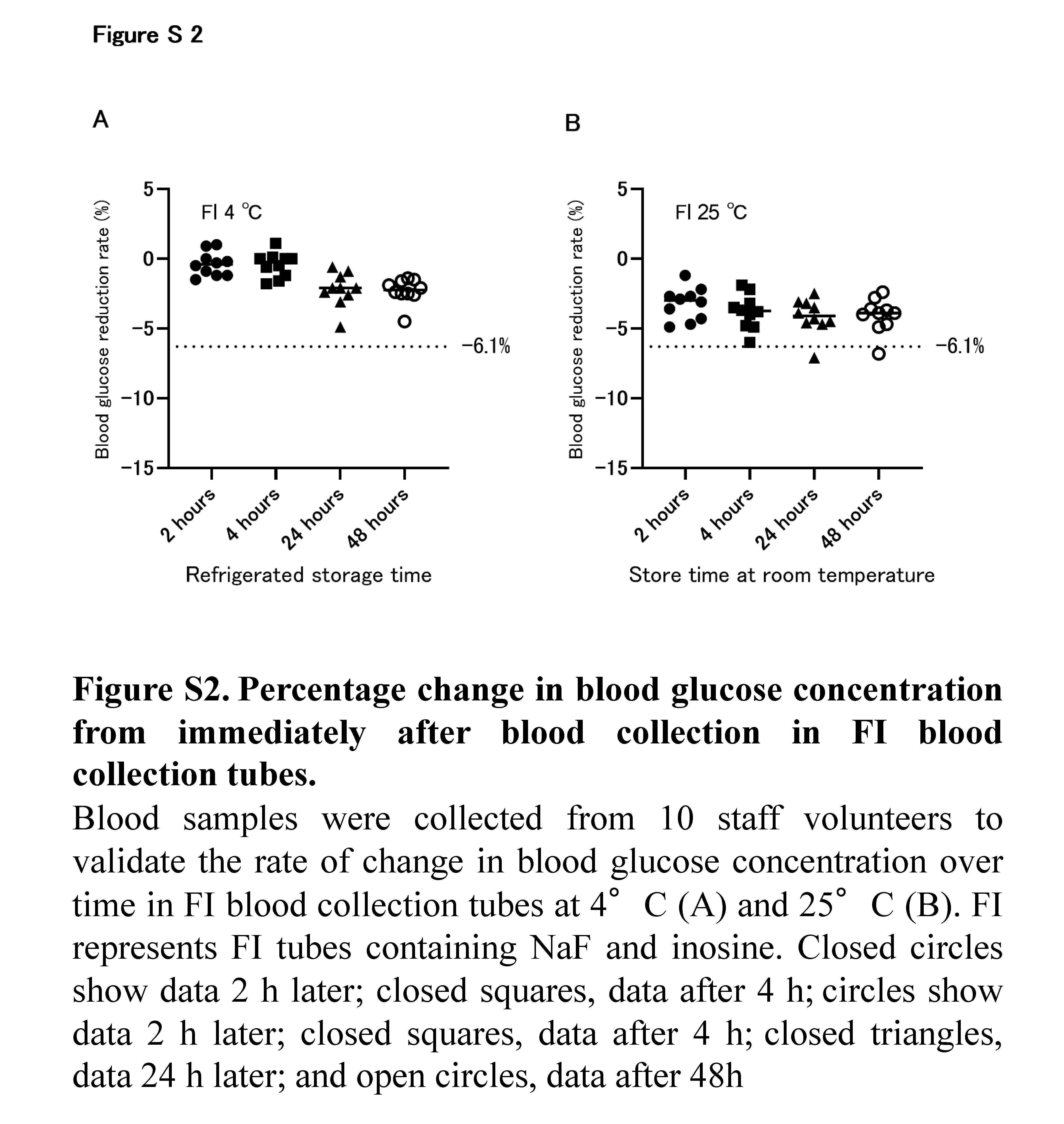

Supplement: Supplementary file 2 — Figure S2: jdb70144‐sup‐0002‐FigureS2.tif. [file JDB-17-e70144-s003.tif]

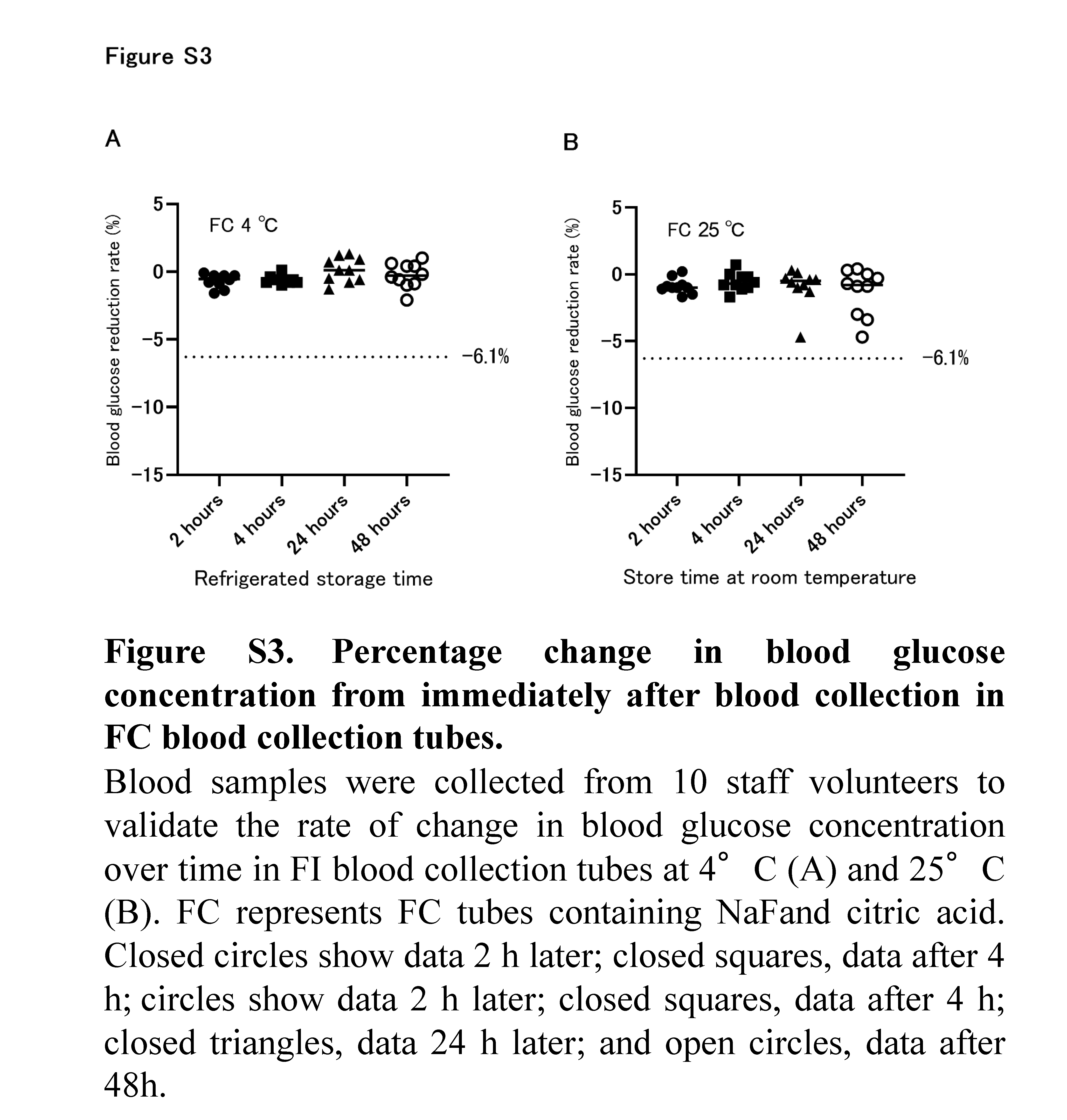

Supplement: Supplementary file 3 — Figure S3: jdb70144‐sup‐0003‐FigureS3.tif. [file JDB-17-e70144-s004.tif]

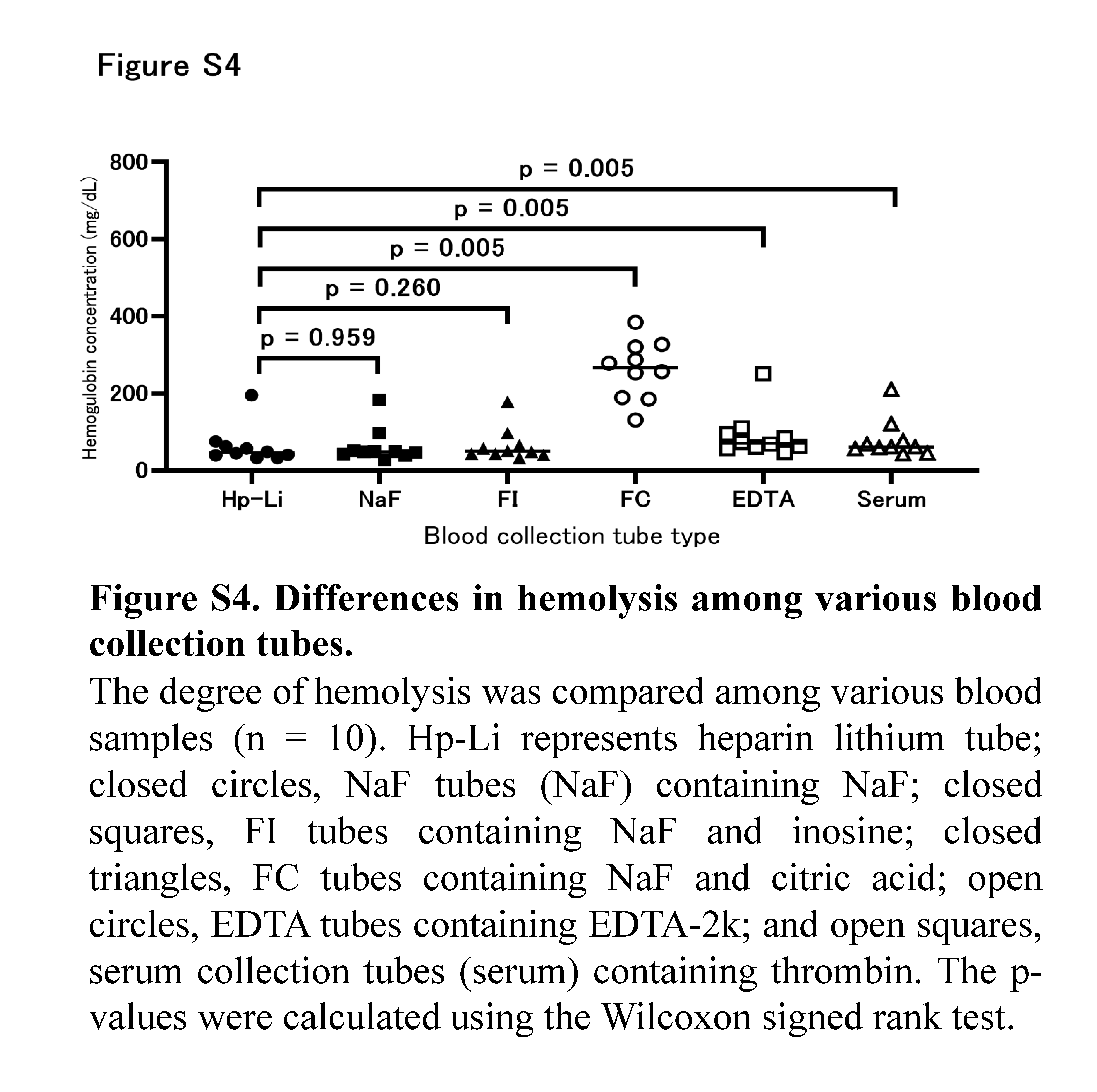

Supplement: Supplementary file 4 — Figure S4: jdb70144‐sup‐0004‐FigureS4.tif. [file JDB-17-e70144-s001.tif]
